# Two-Partner Secretion: Combining Efficiency and Simplicity in the Secretion of Large Proteins for Bacteria-Host and Bacteria-Bacteria Interactions

**DOI:** 10.3389/fcimb.2017.00148

**Published:** 2017-05-09

**Authors:** Jeremy Guérin, Sarah Bigot, Robert Schneider, Susan K. Buchanan, Françoise Jacob-Dubuisson

**Affiliations:** ^1^Laboratory of Molecular Biology, National Institute of Diabetes and Digestive and Kidney Diseases, National Institutes of HealthBethesda, MD, USA; ^2^Molecular Microbiology and Structural Biochemistry, Centre National de La Recherche Scientifique UMR 5086–Université Lyon 1, Institute of Biology and Chemistry of ProteinsLyon, France; ^3^NMR and Molecular Interactions, Université de Lille, Centre National de La Recherche Scientifique, UMR 8576–Unité de Glycobiologie Structurale et FonctionnelleLille, France; ^4^Université de Lille, Centre National de La Recherche Scientifique, Institut National de La Santé et de La Recherche Médicale, CHU Lille, Institut Pasteur de Lille, U1019–UMR 8204–Centre d'Infection et d'Immunité de LilleLille, France

**Keywords:** type V secretion, two-partner secretion, Omp85 transporter, gram-negative bacteria, outer membrane, contact-dependent growth inhibition

## Abstract

Initially identified in pathogenic Gram-negative bacteria, the two-partner secretion (TPS) pathway, also known as Type Vb secretion, mediates the translocation across the outer membrane of large effector proteins involved in interactions between these pathogens and their hosts. More recently, distinct TPS systems have been shown to secrete toxic effector domains that participate in inter-bacterial competition or cooperation. The effects of these systems are based on kin vs. non-kin molecular recognition mediated by specific immunity proteins. With these new toxin-antitoxin systems, the range of TPS effector functions has thus been extended from cytolysis, adhesion, and iron acquisition, to genome maintenance, inter-bacterial killing and inter-bacterial signaling. Basically, a TPS system is made up of two proteins, the secreted TpsA effector protein and its TpsB partner transporter, with possible additional factors such as immunity proteins for protection against cognate toxic effectors. Structural studies have indicated that TpsA proteins mainly form elongated β helices that may be followed by specific functional domains. TpsB proteins belong to the Omp85 superfamily. Open questions remain on the mechanism of protein secretion in the absence of ATP or an electrochemical gradient across the outer membrane. The remarkable dynamics of the TpsB transporters and the progressive folding of their TpsA partners at the bacterial surface in the course of translocation are thought to be key elements driving the secretion process.

## Introduction: an overview of type V secretion

**Figure 1:**
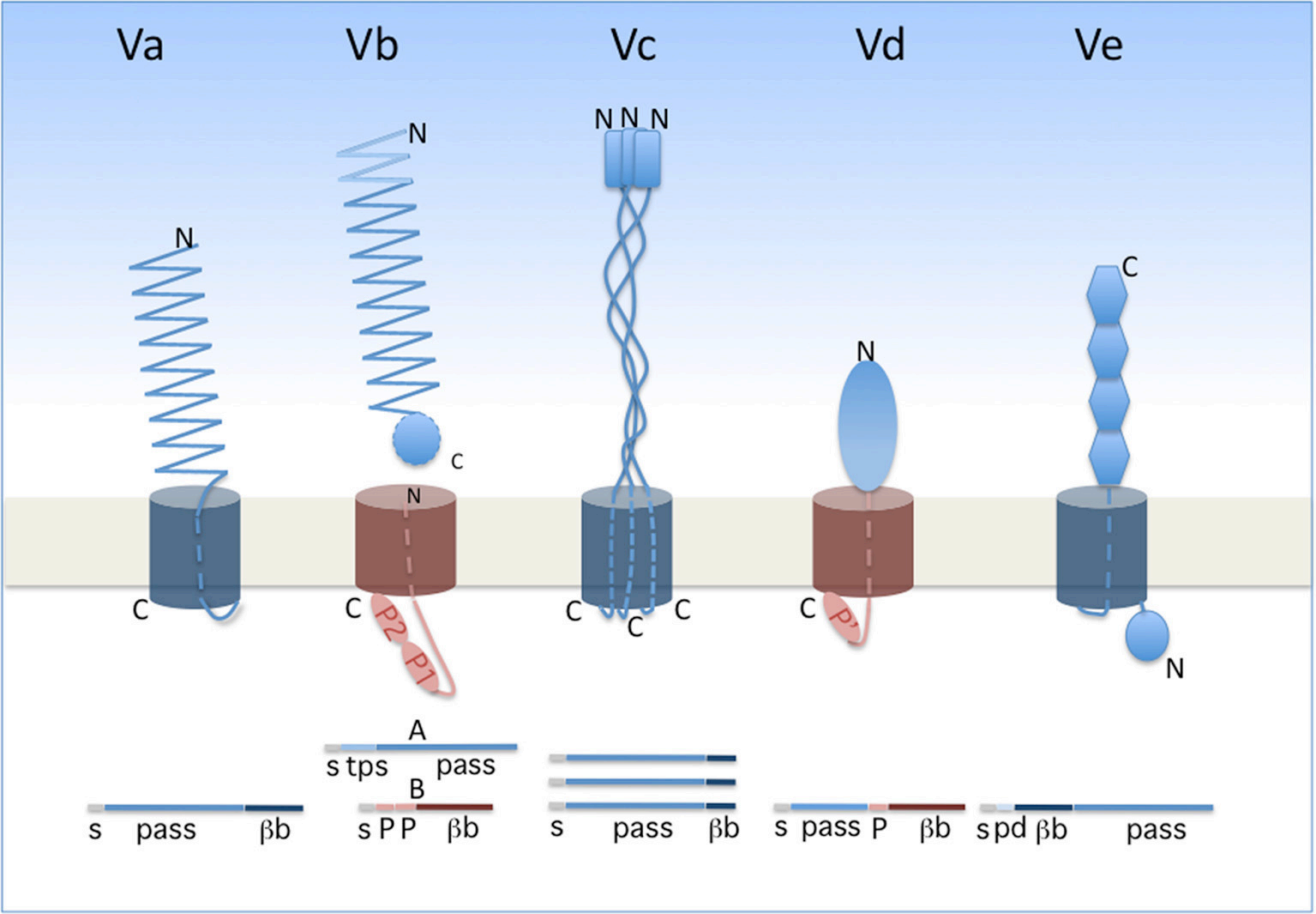
**Type V secretion subtypes**. The proteins involved in each system are represented in their final form, i.e., after completion of secretion. A linear representation is shown underneath the schematics. The two types of β barrels are colored in dark blue (12-stranded barrel) and dark red (16-stranded barrel). The orientation of each protein is indicated by its N and C termini (denoted N and C). The POTRA domains (small ovals) are denoted P1, P2, and P' (the latter being a POTRA-like domain in PlpD). s, signal peptide; pass, passenger domain; βb, β-barrel domain; tps, TPS domain of TpsA proteins; pd, periplasmic domain of type Ve proteins.

Two-Partner secretion (TPS) represents a branch of so-called type V secretion. Widespread in Gram-negative bacteria, type V secretion encompasses several subtypes, identified a–e, that share several general features (Figure 1). The cores of type V systems are composed of one or two proteins. The effector (“passenger”) proteins or domains are transported exclusively across the outer membrane, following Sec-dependent export to the periplasm. In subtypes Va, Vc, Vd, and Ve, the passenger domain is fused to the membrane domain. The latter is necessary and was once thought to be sufficient for secretion of the passenger domain, hence the generic name of “autotransporters” (Pohlner et al., 1987; Leyton et al., 2012). In subtype Vb (i.e., the TPS pathway), in contrast, the passenger protein generically called “TpsA” is separate from its cognate “TpsB” transporter. In the case of TPS systems, thus, a TpsB transporter can have more than one effector, but this does not generally seem to be the case.

The passenger proteins or domains secreted by the type V pathway are generally long and form fibrous structures, often β helices. They frequently contain repeated sequences determining repetitive folds that appear to be specific to the secretion subtype (see below). The transporter components form transmembrane β-barrel pores. In the available X-ray structures, the β barrels are plugged after secretion by one α helix for type Va and Vb systems, three α helices for type Vc systems and an extended polypeptide segment for type Ve systems (Oomen et al., 2004; Meng et al., 2006; Barnard et al., 2007; Clantin et al., 2007; van den Berg, 2010; Fairman et al., 2012; Shahid et al., 2012). No hydrolysable energy source or electrochemical gradient powers type V translocation, and the processes of secretion and folding are thought to be coupled. In line with the latter feature, general properties of the passenger domains include their slow intrinsic folding rate, paucity in Cys residues, high solubility and low propensity to aggregate prior to folding (Junker et al., 2006; Hartmann et al., 2009; Junker and Clark, 2010). Last but not least, a defining feature of the type V pathway is that protein secretion in all subtypes depends on transporters of the Omp85 superfamily (Webb et al., 2012; Heinz and Lithgow, 2014).

In classical autotransporters (ATs; type Va), the passenger domains are mainly adhesins, proteases, or esterases. The same polypeptide contains in succession the passenger domain and the 12-stranded, transmembrane β-barrel domain (Figure 1). Proteolytic processing between the two domains frequently occurs for classical ATs. The prototypical fold of the passenger domain is a β helix, but other structures also occur (Emsley et al., 1996; Otto et al., 2005; van den Berg, 2010). ATs have been the focus of most studies on type V secretion, and they were thoroughly reviewed in several recent articles (Leyton et al., 2012; Grijpstra et al., 2013; van Ulsen et al., 2014).

Trimeric ATs (type Vc) are adhesins that may contribute to biofilm formation of pathogenic bacteria and have no known enzymatic activity (Hoiczyk et al., 2000; Cotter et al., 2005; Kim et al., 2006; Linke et al., 2006; Bentancor et al., 2012). They are homotrimers whose passenger domains assemble into long, rather rigid stalks with a “head,” and harbor domains rich in β structure interspersed with helical coiled coils (reviewed in Fan et al., 2016). Each monomer contributes its four C-terminal β strands to the pore-forming transmembrane β barrel (Leo et al., 2012).

Type Ve proteins are intimins and invasins (Tsai et al., 2010; Leo et al., 2015b; Heinz et al., 2016). In these proteins, the 12-stranded transmembrane β barrel precedes the passenger domain, making them “reverse ATs” (Tsai et al., 2010; Fairman et al., 2012; Oberhettinger et al., 2012). Their modular passenger domains are composed of IgG-like and lectin-like domains. These proteins appear to also have periplasmic extensions involved in dimerization (Leo et al., 2015a).

The little studied type Vd proteins, of which PlpD of *P. aeruginosa* is the prototype, are hybrids between AT and TPS systems. Homologs of PlpD are restricted to specific lineages of Proteobacteria, Fusobacteria, Bacteroidetes, and Chlorobi, suggesting that they may have been acquired by horizontal transfer (Salacha et al., 2010). The passenger protein carries the four sequence blocks typical of patatin-like proteins (PLP) (da Mata Madeira et al., 2016) and has lipolytic activity. It is released into the milieu from the precursor by proteolytic cleavage. The transporter domain, which remains in the outer membrane after maturation of the precursor, is related to TpsB proteins (Salacha et al., 2010).

This short description of type V secretion suggests that new arrangements of the passenger and transporter moieties likely remain to be characterized, which might further expand this broad secretion pathway (Gal-Mor et al., 2008; Arnold et al., 2010; Jacob-Dubuisson et al., 2013). In this review devoted to type Vb secretion, we will first describe the basics of TPS systems. We will then develop functional aspects of the systems involved in contact-dependent growth inhibition (CDI), which thus far appear to be confined to the TPS pathway and are not found in other subtypes of type V secretion. We will cover the structural and mechanistic aspects of the TPS pathway, and to do so we will compare with other Type V systems where relevant. Earlier work will only be mentioned briefly, and thus readers are referred to previous reviews for more details (Jacob-Dubuisson et al., 2013; van Ulsen et al., 2014; Fan et al., 2016).

## TPS systems: generalities

**Figure 2:**
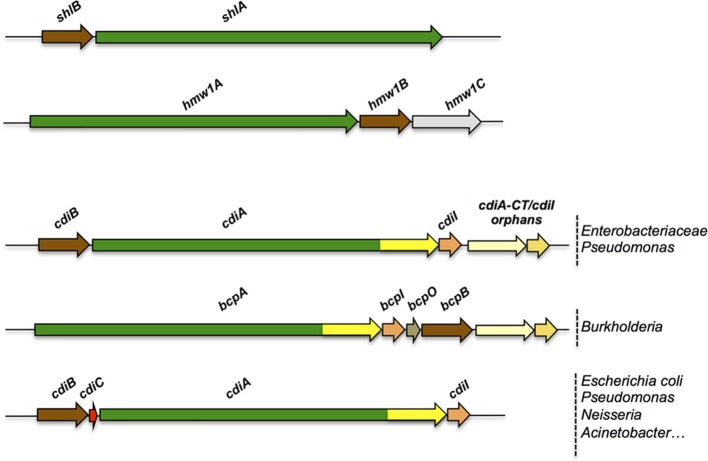
**Organization of TPS operons**. Typical TPS operons are compared with operons coding for CDI systems found in various bacterial genera. Orphan variant *cdiA-CT/cdiI* cassettes are found downstream of some *cdi* operons that likely serve for homologous recombination with full-length *cdiA* genes, thus contributing to the polymorphism of the toxin moieties.

The term Two-Partner Secretion was initially coined to define a distinct secretion pathway exemplified by a handful of systems including ShlAB of *Serratia marcescens*, FhaBC of *Bordetella pertussis*, HpmAB of *Proteus mirabilis*, and HMW1ABC and HMW2ABC of non-typeable *Haemophilus influenzae* (Poole et al., 1988; Schiebel et al., 1989; Domenighini et al., 1990; Uphoff and Welch, 1990; Barenkamp and St Geme, 1994; Willems et al., 1994; Hertle et al., 1999; Jacob-Dubuisson et al., 2001). As implied by the name, the core of a TPS system consists of two proteins, the secretory passenger protein and its transporter across the outer membrane, generically called TpsA and TpsB partners, respectively. In most instances, the genes coding for a TPS system are part of the same operon, but other genetic arrangements have been found (Jacob-Dubuisson et al., 2013) (Figure 2). The degree of specificity of a TpsB transporter for its cognate partner varies between systems, and some TpsBs can secrete more than one TpsA (Julio and Cotter, 2005) or appear to be more promiscuous than others (van Ulsen et al., 2008; ur Rahman and van Ulsen, 2013).

The first TPS systems to be characterized were found to secrete cytolysins or adhesins in pathogenic bacteria (Jacob-Dubuisson et al., 2001, 2004). The list rapidly expanded to include other TPS systems with new or unknown functions in various bacterial genera (Table 1). Later, a whole new group of TPS systems were found to mediate inter-bacterial interactions involving molecular recognition of closely related bacteria and leading to collective—cooperative or competitive behaviors (Aoki et al., 2005, 2010; Willett et al., 2015b). These “contact-dependent growth inhibition” (CDI) systems will be described in more detail below. Insight into these new TPS functions has considerably boosted interest in the field.

**Table 1:** **Diverse functions of TpsA proteins**.

**Class**	**Organism**	**Function**	**Reference**
**CYTOLYSINS/HEMOLYSINS**			
ShlA	*Serratia marcescens*	Cytolysin, hemolysin, pore forming toxin, autophagy induction	Braun et al., 1992; Hertle et al., 1999; Konninger et al., 1999
HpmA	*Proteus mirabilis*	Cytolysin, hemolysin	Uphoff and Welch, 1990; Zwihart and Welch, 1990
EthA	*Edwardsiella tarda*	Cytolysin, hemolysin, host cell adherence, internalization process in fish	Hirono et al., 1997; Strauss et al., 1997; Wang et al., 2010
HhdA	*Haemophilus ducreyi*	Hemolysin	Palmer and Munson, 1995
PhlA	*Photorhabdus luminescens*	Hemolysin	Brillard et al., 2002
ExlA	*Pseudomonas aeruginosa*	Exolysin, plasma membrane rupture of human cells, pore forming toxin	Elsen et al., 2014; Basso et al., 2017
**PROTEASES**			
LepA	*Pseudomonas aeruginosa*	Induction of inflammatory responses trough human protease-activated receptors (PARs)	Kida et al., 2008, 2011
**IRON ACQUISITION**			
HxuA	*Haemophilus influenzae*	Heme acquisition from hemopexin	Cope et al., 1994, 1995
**ADHESINS**			
FHA	*Bordetella pertussis*	Adhesion to epithelial cells, biofilm formation, immunomodulation	Locht et al., 1993; Anderson et al., 2012; Serra et al., 2012
HMW1/HMW2	*Haemophilus influenzae*	Adhesion to epithelial cells	St Geme and Yeo, 2009
EtpA	*Escherichia coli*	Intestinal colonization, adhesion to host cells by binding to the tip of flagella	Roy et al., 2009
EtpB transporter	*Escherichia coli*	Adhesion to epithelial cells	Fleckenstein et al., 2006
CdrA	*Pseudomonas aeruginosa*	Biofilm, binding to Psl exopolysaccharides	Borlee et al., 2010
Ap58 (EnfA) transporter	*Escherichia coli*	Adhesion and hemagglutination activities	Monteiro-Neto et al., 2003
**CDI SYSTEMS**			
CdiA	Enterobacteria species, *Pseudomonas aeruginosa*	Contact dependent growth inhibition, biofilm	Aoki et al., 2005; Ruhe et al., 2015; Mercy et al., 2016
BcpA	*Burkholderia* species	Contact dependent growth inhibition, autoaggregation, biofilm, kin recognition, contact-dependent signaling	Anderson et al., 2012, 2014; Garcia et al., 2013, 2016
HecA^*^	*Dickeya dadantii*	Adhesin, attachment and aggregation on leaves, killing of epidermal cells	Rojas et al., 2002
HrpA^*^	*Neisseria meningitidis*	Adhesin, binding to epithelial cells, intracellular escape, immune evasion, biofilm formation	Schmitt et al., 2007; Tala et al., 2008; Neil and Apicella, 2009
HxfA^*^, HxfB^*^	*Xylella fatidiosa*	Autoaggregation	Guilhabert and Kirkpatrick, 2005
XacFhaB^*^	*Xanthomonas axonopodis*	Adhesin, biofilm formation	Gottig et al., 2009
FhaB^*^	*Xanthomonas fuscans*	Vascular transmission to bean seeds	Darsonval et al., 2009
MchA^*^	*Moraxella catarrhalis*	Adhesin, binding to epithelial cells	Plamondon et al., 2007
MhaB^*^	*Moraxella catarrhalis*	Adhesin, binding to epithelial cells	Balder et al., 2007
AbFhaB^*^	*Acinetobacter baumannii*	Adhesin, binding to epithelial cells and fibronectin	Perez et al., 2016
**UNKNOWN FUNCTION**			
RscA	*Yersinia enterolitica*	Limitation of splenic dissemination	Nelson et al., 2001
LspA1/LspA2	*Haemophilus ducreyi*	Inhibition of phagocytosis	Dodd et al., 2014
OtpA	*Escherichia coli*	Unknown	Choi et al., 2007
PdtA	*Pseudomonas aeruginosa*	Virulence in nematode model	Faure et al., 2014
BpaA	*Burkholderia pseudomallei*	Unknown	Brown et al., 2004
HlpA	*Pseudomonas putida*	Seed and Root colonization	Molina et al., 2006
PfhB1, PfhB2	*Pasteurella multocida*	Virulence in septicemic mouse model	Fuller et al., 2000

* *Predicted CDI system based on genetic analysis*.

Thus, more and more TPS systems have been characterized over the years, overwhelmingly in pathogens. Whether this reflects their actual distribution in Gram-negative bacteria is very much in doubt, as pathogenic bacteria are much more extensively studied than environmental bacteria. Actually, the discovery of CDI systems and of their roles in collective behaviors of bacteria rather suggests that TPS systems are likely to be widely distributed, well beyond bacterial pathogens (Jacob-Dubuisson et al., 2013).

## Old and new functions of TPS systems

### Functions of TPS systems in bacterial pathogens

In pathogenic bacteria, TpsA cytolysins/hemolysins are probably rather common (Table 1). Orthologs of the prototypic ShlA and HpmA proteins have been found in various genera. In other cases the specific activities of the TpsA proteins have not necessarily been determined. In many instances, the TPS operons are up-regulated upon bacterial entry into the host, and experimental cellular and animal models of infection indicate their importance in host-pathogen interactions. Examples include the RscA protein of *Yersinia enterocolitica* that limits systemic dissemination (Nelson et al., 2001), and the TpsA proteins PfhB1 and B2 of *Pasteurella multocida* and PdtA of *P. aeruginosa* that were shown to contribute to virulence in models of mouse septicemia and *Caenorhabditis elegans* infection, respectively (Fuller et al., 2000; Faure et al., 2014).

Specific functions have nevertheless been ascribed to several TpsA proteins. Thus, some are involved in iron acquisition, including HxuA of *H. influenzae* (Fournier et al., 2011) and possibly HlpA in the root-colonizing bacterium *Pseudomonas putida* KT2440 (Molina et al., 2006). At the surface of the cell, HxuA interacts with hemopexin, a serum glycoprotein involved in heme recycling. This interaction causes release of the heme from hemopexin, making it available for import by the dedicated HxuC receptor (Fournier et al., 2011). In the case of enterotoxigenic *Escherichia coli* (ETEC), the TpsA protein EtpA is necessary for intestinal colonization. By binding to the tips of flagella, EtpA mediates bacterial adherence to intestinal cells (Roy et al., 2009). The TpsA proteins LspA1 and LspA2 of *H. ducreyi* are required for full virulence of this pathogen by inhibiting phagocytosis through the phosphorylation of the eukaryotic Src tyrosine kinase (Vakevainen et al., 2003; Ward et al., 2003; Dodd et al., 2014). In *P. aeruginosa*, the secreted TpsA protein LepA is a protease that activates the inflammatory response (Kida et al., 2008). Finally, the TpsB protein EtpB and its homolog EnfA, renamed Ap58, of enteroaggregative *E. coli* AEC O111H12 are involved in the adherence and hemagglutination activities of these isolates (Monteiro-Neto et al., 2003; Fleckenstein et al., 2006).

### CDI systems

**Figure 3:**
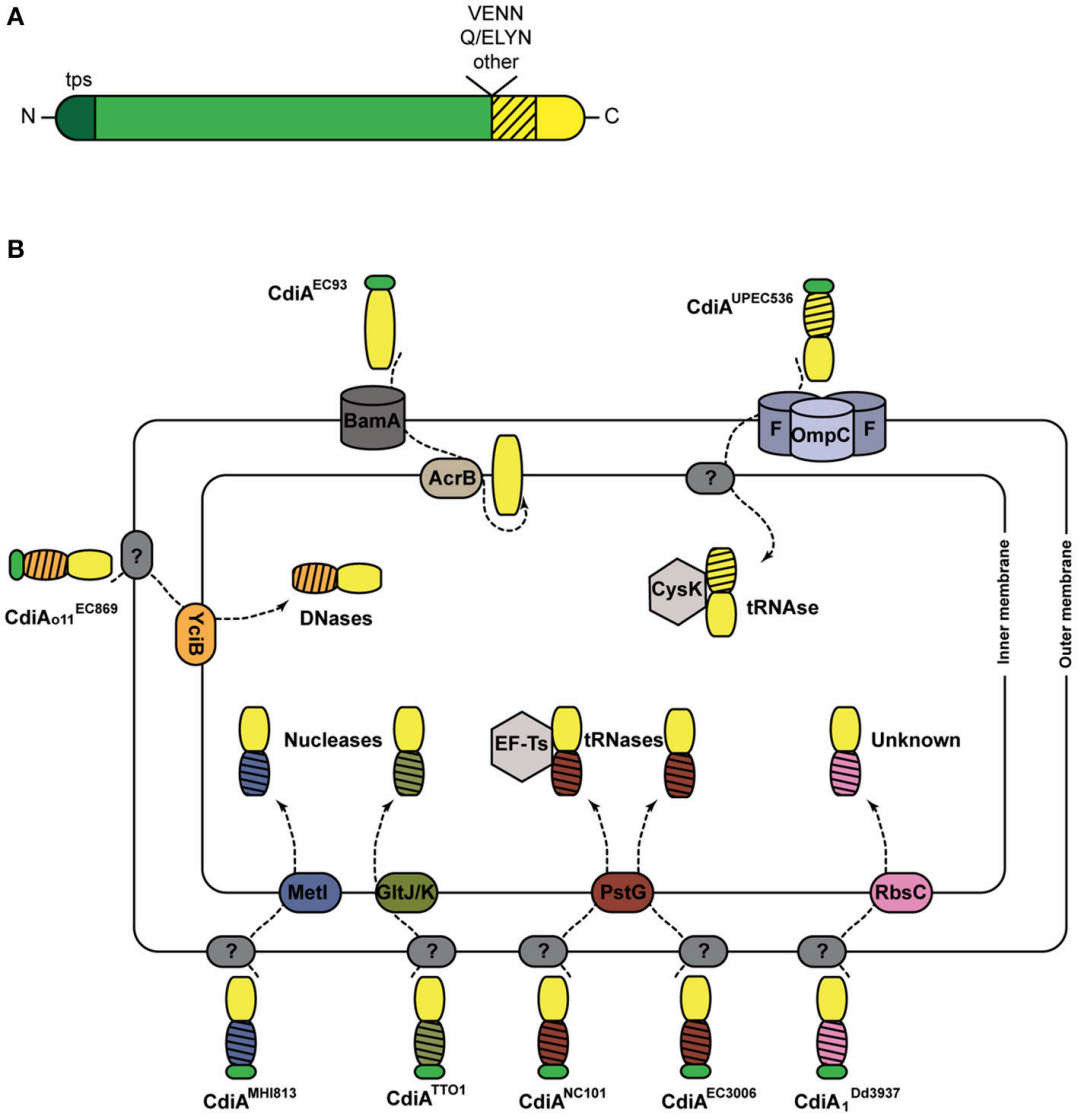
**CDI systems. (A)** Linear representation of a typical CdiA protein. In addition to the TPS domain (in darker shade of green), found in all TpsA proteins and necessary for secretion, CdiA proteins also harbor specific sequence motifs such as VENN or (Q/E)LYN that separate the long central region from the CdiA-CT domain. The first part of the CT domain (hatched) is necessary for import into the target cell, while the second part carries the toxic activity of the protein. **(B)** Cell entry pathways of CdiA-CT. The pre-toxin motif that demarcates the variable CdiA-CT is represented in green. The receptors of CdiA-CT domains at the surface of the target bacteria remain unknown in most cases (indicated by ?). CdiA-CT^EC93^ of *E. coli* EC93 is predicted to be a pore-forming domain that inserts into the inner membrane. For the other CdiA-CT domains, their N-terminal domains (hatched) are colored according to the membrane proteins putatively involved in their entry into the cytoplasm. The tRNase activities of CdiA-CT^UPEC536^ and CdiA^NC101^ are activated by the cytoplasmic cysteine synthase A CysK and the translational elongation factor EF-Ts, respectively. The CdiA toxin of *E. coli* EC869 is a DNase, and *E. coli* NC101 and EC3006 produce tRNases. The CdiA-CT moieties of *E. coli* MHI813 and *Photorhabdus luminescens* TTO1 harbor conserved nuclease_NS2 and endonuclease_VII domains, respectively. The toxic activity of CdiA produced by *Dickeya dadantii* 3937 is unknown.

First described in the *E. coli* EC93 strain isolated from rats, bacterial growth inhibition of neighboring cells was revealed to occur by direct interactions and to imply a specific TPS system which was renamed CDI (Aoki et al., 2005). Growth inhibition is mediated by toxic activities associated with the last ~300 C-terminal residues of the CdiA proteins, called CdiA-CT (Figure 3A). Upon cell contact, the surface-exposed CdiA binds the target cell, and its CdiA-CT is delivered, most likely after being cleaved off the rest of the protein, into the target bacterium. CDI^+^ cells produce an immunity protein, CdiI, which protects them from fratricide by interacting with the cognate CdiA-CT and blocking its toxic activity. CDI systems are widespread among α, β, and γ Proteobacteria (Ruhe et al., 2013), but the genetic organization of those systems differs between species. Most of them share the classical *cdiBAI* locus found in *E. coli* EC93 (Aoki et al., 2005), but the *cdi* genes of *Burkholderia* species constitute a unique class with the distinct *bcp* (*Burkholderia* CDI proteins) nomenclature. This atypical *bcpAIB* gene organization can also comprise an accessory *bcpO* gene between *bcpI* and *bcpB* (Anderson et al., 2012) (Figure 2). Depending on the species, it codes for a predicted periplasmic lipoprotein or for a cytoplasmic protein of unknown function, which is necessary for the function of BcpA (Anderson et al., 2012). In addition, orphan variant *cdiA-CT/cdiI* cassettes are found downstream of the *cdi* operons in some species that likely serve for homologous recombination with full-length *cdiA* genes contributing to the variability of the CdiA-CT domain between CdiA proteins. Finally, several Proteobacteria contain a *cdiBCAI*-type operon, with *cdiC* encoding a protein predicted to belong to the toxin acyltransferase family (Ruhe et al., 2013; Ogier et al., 2016) (Figure 2). The function of the CdiC protein is unknown, but it might be involved in CdiBA biogenesis and/or post-translational modifications of CdiA.

Like TpsA proteins, CdiAs are composed of large regions rich in β structure, including the N-terminal TPS domain. They however differ from typical TpsA proteins in their C termini (Figure 3A). Sequence alignments have indicated that CdiA proteins share conserved C-proximal motifs of unknown function, such as (Q/E)LYN in *Burkholderia*, VENN in other bacteria or yet other motifs in some *Pseudomonas* (Aoki et al., 2005; Anderson et al., 2012; Mercy et al., 2016) (Figure 3A). The VENN motif of CdiA^UPEC536^ was shown not to be required for toxin import in the target cell (Beck et al., 2014a). One hypothesis is that the conserved motifs might be involved in the proteolytic processing of the CdiA proteins to release their C-terminal domains, as for the bacterial intein-like (BIL) domain of CdiA of *P. syringae* (Amitai et al., 2003). Beyond the conserved motifs, the sequences diverge abruptly, and therefore the so-called “CdiA-CT” domains are highly variable. They have been identified as the toxic domains, carrying DNase, tRNase, or pore-forming activities (Aoki et al., 2010; Zhang et al., 2011; Ruhe et al., 2013; Webb et al., 2013).

The toxins need to reach the cytosol of target cells by crossing both bacterial membranes (Figure 3B). The study of their translocation pathways has revealed complex mechanisms that do not seem to be conserved among CDI systems. They have mostly been investigated in Enterobacteria, and a summary of these findings is depicted in Figure 3B. The CdiA proteins must bind outer membrane receptors before their CdiA-CT moieties are translocated into the target cells. Only two such receptors have been identified to date, BamA for CdiA^EC93^ and heterotrimeric osmoporins composed of OmpC and OmpF for CdiA^UPEC536^ (Aoki et al., 2008; Beck et al., 2016). The current model proposes that after recognition, proteolytic cleavage releases the CdiA-CT from the full CdiA protein through an unknown mechanism. The CdiA-CT is translocated into the periplasm, where it recognizes an inner membrane receptor. Import across the cytoplasmic membrane exploits specific membrane proteins, and the N-terminal part of the CdiA-CT domain plays a crucial role in this step (Willett et al., 2015a) (Figure 3B). Once inside the cell, the CdiA-CT domains deploy their toxic activity, which is dictated by their sequences and their folds (Zhang et al., 2011). Activation of nuclease activity generally depends on host factors. The O-acetylserine sulfhydrylase CysK and the elongation factor Ts (Ef-Ts) are required for the activities of CdiA-CT^UPEC536^ and CdiA^NC101^, respectively (Diner et al., 2012; Kaundal et al., 2016; Jones et al., 2017).

The observations that CDI systems mostly target closely related species and that CdiA proteins preferentially bind kin receptors, thus allowing interactions between self-bacteria, suggest a role for CDI systems beyond competition in collective behaviors of bacteria. This hypothesis has recently been confirmed in *Burkholderia*, which use CDI proteins to establish signaling between CDI^+^ neighbors and thus to affect gene expression in immune recipient bacteria (Garcia et al., 2016). This “contact-dependent signaling” (CDS) implies a functional CdiA-CT domain, but it is restricted to specific toxic domains. In line with signaling functions, some CDI systems help to retain genetic elements through a surveillance mechanism (Ruhe et al., 2016). This property is consistent with the fact that most *cdi* genes are typically found on genomic or pathogenicity islands (Klee et al., 2000; Dobrindt et al., 2002; Tuanyok et al., 2008; Tumapa et al., 2008; Siddaramappa et al., 2011; Willett et al., 2015b; Ruhe et al., 2016). The maintenance of genetic material by CDI is linked to toxin exchange between sibling cells and might serve to maintain the *cdi*-containing islands in a population.

Many TpsA adhesins have now been redefined as CdiA proteins (Table 1). Some of them contribute to the virulence of necrogenic bacterial phytopathogens, including *Dickeya dadantii* (Rojas et al., 2002), *Xylella fastidiosa* (Guilhabert and Kirkpatrick, 2005), and *Xanthomonas axonopodis* (Gottig et al., 2009), with activities ranging from adherence to biofilm formation to epidermal cell killing. Interestingly, some CdiA proteins appear to mediate biofilm formation, but in a CDI-independent manner (Garcia et al., 2013; Ruhe et al., 2015). Whether TpsA proteins might generally be multifunctional remains an open question, but this would be quite conceivable given their large sizes.

## Regulation of *tps* expression

Although *tps* genes appear to be expressed during infection and no specific regulatory pathways have been ascribed to this regulation, several factors modulating TPS production have been characterized (Table 2). To date, the two-component system BvgAS is the best-studied regulatory pathway, controlling *fhaB* and *fhaC* gene expression of *B. pertussis* (Scarlato et al., 1991). Other signal transduction systems have been identified as regulators of *tps* operons in several bacterial species. Quite frequently, the mechanism of production is closely related to the function of the TpsA protein. Thus, a large number of TPS systems are produced upon iron limitation, such as HxuABC involved in iron acquisition (Wong and Lee, 1994; Cope et al., 1995), or cytolysins/hemolysins whose production in low-iron conditions might be a means for the bacteria to release iron from eukaryotic cells or erythrocytes. In *P. aeruginosa*, the intracellular level of cyclic diguanylate monophosphate (c-di-GMP) controls the *cdrAB* operon (Borlee et al., 2010) through the transcriptional regulator FleQ (Baraquet and Harwood, 2015). In addition, the PhoBR two-component system and PUMA3 cell-surface signaling, both activated upon phosphate starvation, positively control the production of the PdtAB system (Llamas et al., 2009; Faure et al., 2014). Finally, two CDI systems are regulated by the post-transcriptional regulator RsmA (Mercy et al., 2016).

**Table 2:** **Factors involved in the regulation of TPS operons**.

***tps* genes**	**Organism**	**Regulation**	**Reference**
*hxuABC*	*Haemophilus influenzae*	Iron-limitation, Fur regulator	Wong and Lee, 1994; Cope et al., 1995
*ethBA*	*Edwarsiella tarda*	Iron-limitation, Fur and EthR regulators, EsrAB two-component system	Hirono et al., 1997; Wang et al., 2009, 2010
*phlBA*	*Photorhabdus luminescens*	Iron-limitation	Brillard et al., 2002
*shlBA*	*Serratia marcescens*	Iron-limitation, RcsB regulator, RssAB two-component system	Poole and Braun, 1988; Lin et al., 2010; Di Venanzio et al., 2014
*etpBAC*	*Escherichia coli*	Iron-limitation	Haines et al., 2015
*rscBAC*	*Yersinia enterolitica*	RscR regulator	Nelson et al., 2001
*cdrAB*	*Pseudomonas aeruginosa*	c-di-GMP level, FleQ regulator	Borlee et al., 2010; Baraquet and Harwood, 2015
*fhaB*-*fhaC*	*Bordetella pertussis*	BvgAS two-component system	Scarlato et al., 1991
*lspB*-*lspA2*	*Haemophilus ducreyi*	CpxRA two component system, Fis protein	Labandeira-Rey et al., 2009, 2013
*pdtB*-*pdtA*	*Pseudomonas aeruginosa*	Phosphate limitation, PhoBR two-component system, PUMA3 system	Llamas et al., 2009; Faure et al., 2014
*cdiBAI*	*Pseudomonas aeruginosa*	RsmA regulator	Mercy et al., 2016

## Additions to the core: other proteins involved in TPS systems

A number of TPS systems comprise more than two partners (Figure 2). This is typically the case for the *cdiI, cdiO, cdiC* genes of CDI systems. In non-typeable *H. influenzae*, TPS systems are encoded by three-gene operons, *hmw1abc* and *hmw2abc* (Barenkamp and St Geme, 1994). *hmw1c* and *hmw2c* code for cytoplasmic glycosyltransferases. HMW1C covalently modifies Asn residues in repeated sequences of HMW1 with mono- and di-hexoses, which increases the stability of the adhesin against proteolytic degradation and antagonizes its release from the cell surface (Grass et al., 2003, 2010; Gross et al., 2008). Other TpsA proteins are also glycosylated (Fleckenstein et al., 2006), as are the passenger domains of a subset of proteins secreted by the type Va pathway (Benz and Schmidt, 2001; Moormann et al., 2002; Charbonneau et al., 2007; Charbonneau and Mourez, 2008; Côté et al., 2013). In addition to slowing down proteolytic degradation, these modifications appear to modify the properties of the adhesins and to modulate their secretion.

The hemopexin TpsA protein HxuA of *H. influenzae* is part of a three-gene operon, in which *hxuB* codes for the TpsB transporter and *hxuC* for a TonB-dependent transporter (Cope et al., 1995; Fournier et al., 2011). All three proteins are necessary to capture heme bound to hemopexin as an iron source. After binding hemopexin, HxuA would trigger heme release for its capture by HxuC (Zambolin et al., 2016).

Finally, some TpsA proteins undergo proteolytic maturation in the course of secretion, but the genes coding for their putative maturation proteases appear not to be part of the TPS-coding operons. Thus, the mature FHA adhesin of *B. pertussis* results from several proteolytic maturation steps (Coutte et al., 2001; Mazar and Cotter, 2006; Melvin et al., 2015). The protease involved in the release of the mature FHA protein from the cell surface is a subtilisin autotransporter called SphB1 (Coutte et al., 2001, 2003). Although no other substrates are known for SphB1, its gene does not belong to the locus encompassing *fhaB* and *fhaC*. Nevertheless, all three are regulated in a coordinated fashion, being part of the virulence regulon of *B. pertussis* (Antoine et al., 2000). In *P. aeruginosa*, the adhesin CdrA is a substrate of the periplasmic protease LapG whose activity is controlled by the intracellular level of c-di-GMP (Cooley et al., 2015; Rybtke et al., 2015). At low c-di-GMP level, LapG cleaves the periplasmic C-terminal tail of CdrA and releases this adhesin from the cell surface to prevent CdrA-dependent biofilm formation. Yet unidentified proteases are involved in the maturation of other TpsA proteins (Ward et al., 1998; Grass and St Geme, 2000; Schmitt et al., 2007).

## Anatomy of TPS systems

### TpsA proteins

TpsA proteins are large exoproteins (~100–650 kDa) that progressively fold at the cell surface in the course of secretion across the outer membrane (see below). In spite of their different functions, all TpsA proteins harbor long stretches of imperfect repeats and are predicted to have high contents of β strand structure organized in fibrous, β-helix folds (Kajava and Steven, 2006a). First observed in pectate lyase (Yoder et al., 1993), the β-helix structure is a solenoid composed of long, parallel β sheets along the axis of the helix (Kajava et al., 2001; Kajava and Steven, 2006b). Its overall shape is provided by the stacking of coils, each of which is composed of three short β strands and forms a complete turn of the β helix. Each β strand interacts via hydrogen bonds with the closest β strands from previous and following coils, forming long parallel β sheets along the axis of the molecule. The interior of a β-helix protein is tightly packed with mostly hydrophobic residues, resulting in a very stable fold (Kajava and Steven, 2006a). Analysis of available structures has also highlighted structural elements that project out of the β-helical core and might carry out specific functions. The notion that the β helix serves as a scaffold to present functional loops or domains at a distance from the bacterial surface does not preclude the possibility that it also has specific functions of its own, such as mediating homotypic interactions that may contribute to biofilm formation (Menozzi et al., 1994; Ruhe et al., 2015).

The structural study of TpsA proteins is complicated by their large size and poor solubility. The fact that they must be secreted by their specific partners to acquire their native fold is a limiting factor for overexpression. Using X-ray crystallography, the majority of structures solved thus far are N-terminal TpsA fragments containing the “TPS” domain, or C-terminal fragments associated with toxin activities of CDI systems. One full-length TpsA structure has been solved, that of HxuA from *H. influenzae* (Zambolin et al., 2016).

**Figure 4:**
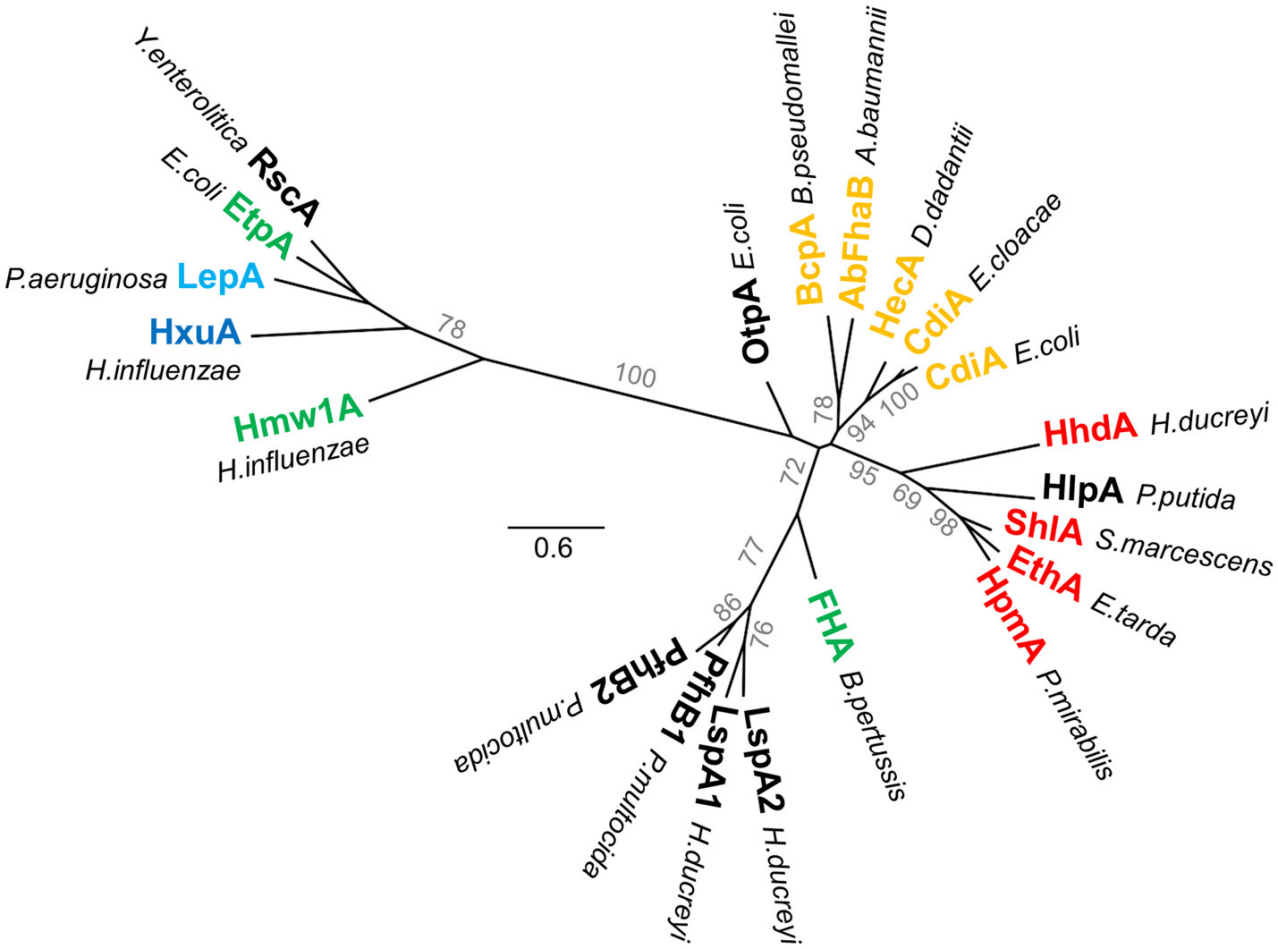
**Phylogenetic tree of TPS domains**. Phylogenetic tree of TPS domains of TpsA proteins cited in this review. The tree shows the subdivision of TpsA proteins into two different families. The proteins also globally form clusters according to function (red for cytolysins/hemolysins, green for adhesins, orange for CDI systems, blue for proteases, dark blue for iron acquisition). The limits of the TPS domains included in the analysis were defined using both sequence similarities and secondary structure predictions, with available X-ray structures used as references. The amino acid sequences were aligned using Promals3D (Pei et al., 2008). PhyML implemented in Geneious v7.1.2 was used to generate an unrooted phylogenetic tree, where scale bars represent the number of substitutions per site, and bootstrap values above 50 (percentages of 1,000 replicates) are shown next to the branches.

All TpsA proteins share a conserved, ~250-residue TPS domain, a.k.a. the secretion domain, corresponding to the minimal region needed for secretion. Located at the N terminus of the mature protein, this essential region mediates molecular recognition of the TpsB transporter in the periplasm, while coupling secretion and folding at the surface of the cell (see below) (Schönherr et al., 1993; Uphoff and Welch, 1994; Renauld-Mongénie et al., 1996; Jacob-Dubuisson et al., 1997; Grass and St Geme, 2000; Clantin et al., 2004; Surana et al., 2004; Hodak et al., 2006; Yeo et al., 2007; Weaver et al., 2009). Two subfamilies of TPS domains have been identified on the basis of sequence alignments (Yeo et al., 2007; Jacob-Dubuisson et al., 2009) and appear distinctly in the phylogenetic tree of TPS domains (Figure 4) (Yeo et al., 2007; Jacob-Dubuisson et al., 2009). In addition, subgroups can be defined based on TpsA functions, probably reflecting specificities in the steps of recognition and secretion initiation for these different groups. Thus far, four structures containing TPS domains are available, two of the first family, Fha30 of *B. pertussis* (Clantin et al., 2004) and HpmA265 of *P. mirabilis* (Weaver et al., 2009), and two of the second family, Hmw1-PP (*H. influenza;* Yeo et al., 2007), and HxuA of *H. influenzae* (Baelen et al., 2013; Zambolin et al., 2016).

**Figure 5:**
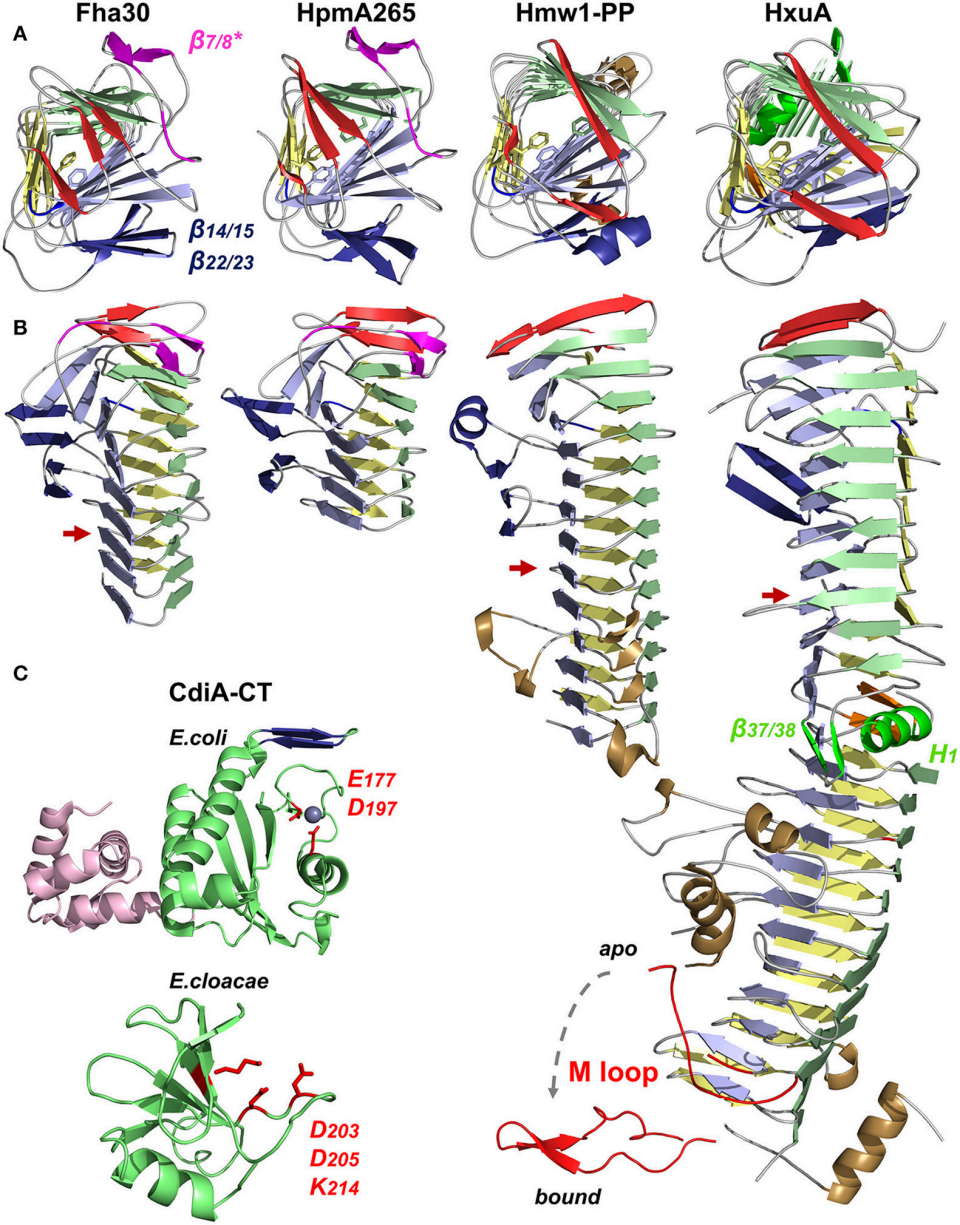
**X-ray structures of TpsA proteins**. Cartoon representations of Fha30 (PDB entry 1rwr), HpmA265 (3fy3), Hmw1-PP (2odl), HxuA (4mr6 and 4rt6), CdiA-CT of *E. coli* EC869 (4g6u), and CdiA-CT of *E. cloacae* (4ntq). The parallel β sheets PB1, PB2, and PB3 are colored in light green, blue, and yellow, respectively. The first β strands corresponding to the N-terminal cap are represented in red. The NPNG motif and the extra-helical β-sheet β_14/15_–β_22/23_ conserved among TpsA proteins are in blue, and specific elements from the FHA subfamily (the NPNL motif and β_7/8_^*^) are in magenta. To harmonize the nomenclature between proteins, the numbers given to the structural elements may differ from those in the original publications. **(A)** Views from the N-terminal top of the β-helix axis. Residues of the aromatic cluster are shown in stick representation. **(B)** Side view of TpsA structures. Red arrows indicate the end of the TPS domain. Of note, β_14_ is replaced by an α helix in Hmw1-PP, and part of that β sheet is missing in full-length HxuA. In these two structures, the extra-helical elements in the C-terminal moiety are in brown. For HxuA, structural elements responsible for the twist in the middle of the β helix are highlighted in green (α helix H1 in PB1, and β hairpin β_37_/β_38_). The M loop (in red) is represented in two conformations, with and without hemopexin, denoted bound and apo, respectively. **(C)** Structures of the CdiA-CT domains of CdiA proteins. The nuclease domain is highlighted in green, with the side chains of the active site residues in red. For the CdiA-CT of *E. coli*, the active-site Zn^2+^ ion is shown as a sphere, and the N-terminal α-helical bundle is colored in pink. The β hairpin involved in forming β-sheet structure with the immunity protein is in blue. The structures of the toxins were solved in complex with their respective immunity proteins (not represented), arguing that the CdiA-CT moieties must be in inactive conformations.

TpsA proteins form right-handed β helices with three parallel β sheets, referred to as PB1, PB2, and PB3 (Figure 5A). The N-terminal β strands cap the β-helix tip by protecting the hydrophobic core from the solvent. The interior of the first β-helix coil is stabilized by a cluster of conserved aromatic residues from β_4_, β_5_, and β_6_ (the nomenclature used here for strand numbering is that proposed in Baelen et al., 2013) (Figure 5A) (Clantin et al., 2004). Thereafter, the coils become more regular to eventually display a triangular-shaped cross-section in the C-terminal moiety of the TPS domain (Figure 5B). TPS domains present structural elements protruding from the β-helical core. Thus, in the middle of the TPS domain, an anti-parallel β sheet forms a β-sandwich structure with the core of the β helix (Figure 5B). In Hmw1-PP, the β sheet is formed by three β strands, with an α helix replacing the fourth one. TpsA proteins also contain a highly conserved NPNG motif which forms a β turn between strands β_10_ in PB2 and β_11_ in PB3 and is crucial for folding and secretion (Jacob-Dubuisson et al., 1997; St Geme and Grass, 1998; Grass and St Geme, 2000; Hodak et al., 2006). Close to the N-terminus and only shared by Fha30 and HpmA265, a second anti-parallel β hairpin (denoted β_7/8_^*^) that harbors a conserved motif, NPNL, is also important for secretion (Schönherr et al., 1993; Jacob-Dubuisson et al., 1997; Hodak et al., 2006). As this anti-parallel β hairpin is absent from the second family of TPS domains, it is not essential for TpsA proteins in general but probably reflects specific secretion and/or folding properties of the TPS domains in the first family (Figure 4).

Due to their large size, little is known about the structural organization of full-length TpsA proteins beyond their TPS domains. Electron microscopy has been used to describe the global shape of 500-Å-long FHA (Makhov et al., 1994; Kajava et al., 2001). Based on electron microscopy and X-ray data, the most likely model for full-length FHA is that of a straight, elongated β helix formed from long regions of tandem repeats. The adherence functions of the protein likely map to extended loops and extra-helical motifs along the mature helix. A non-repeat domain of unknown structure that follows the β helix is also important for function (Kajava et al., 2001; Melvin et al., 2015). Folding of this domain requires the non-secreted C-terminal portion of the FHA precursor that is processed to form the mature protein and controls its release (Noel et al., 2012; Melvin et al., 2015).

The structure of the relatively small TpsA protein HxuA (96.3 kDa, 120 Å long) is shown in Figure 5B. HxuA is a right-handed β helix whose N-terminal and C-terminal moieties have different orientations because of a kink in PB1. This twist in the middle of the β helix divides the protein in two segments: the N-terminal moiety with the TPS domain, and the C-terminal moiety associated with HxuA function (Figures 5A,B). The latter is highly asymmetric due to α helices and long loops, including the essential M loop, that protrude from the β-helical core. The last residues resolved in the structure form an amphipathic α helix whose hydrophobic face interacts with PB1 while the other is solvent-exposed (Figure 5B).

Analysis of the HxuA-hemopexin complex by transmission electron microscopy and X-ray crystallography has revealed that hemopexin interacts only with the C-terminal moiety of HxuA (Zambolin et al., 2016). The interaction with hemopexin induces a large-scale motion of the M loop (Figure 5B) which then forms polar interactions with residues in the heme-binding pocket of hemopexin that trigger heme release.

In the HxuA structure, the 66 C-terminal amino acids are not detected in the electron density map. Similar to that of HMW1, the C terminus of HxuA is expected to remain in the periplasm. This anchor domain harbors a disulfide bond, which creates a globular loop region that locks the TpsA C terminus in the pore of its TpsB partner (Buscher et al., 2006). Preventing HxuA release into the supernatant must be important to couple heme release and import. The presence or absence of this C-terminal anchor domain possibly reflects functional differences among TpsA proteins.

To date, no full structure has been reported of the very large CdiA proteins (180–650 kDa), but several structures of CdiA-CT domains have been obtained by producing the toxic domain in complex with its cognate CdiI immunity protein. For CdiA-CT of *E. coli* EC869, the structure starts ~80 residues after the VENN motif. The N-terminal and C-terminal domains of CdiA-CT form a 4-helix bundle and a central β sheet sandwiched between four α helices, respectively (Figure 5C). The second domain is structurally similar to type IIS restriction endonucleases, with a Zn^2+^ ion in the active site (Kachalova et al., 2008; Morse et al., 2012). Adjacent to the active site, a β hairpin interacts with the immunity protein by β augmentation, forming an anti-parallel β sheet (Morse et al., 2012). The structure of another CdiA-CT corresponding to the last 75 amino acids of the *E. cloacae* CdiA protein in interaction with its immunity protein shows a completely different fold, displaying structural homology with the nuclease domain of colicin E3. The toxin domain is folded into one α helix followed by a five-stranded anti-parallel β sheet and is expected to cleave 16S rRNA (Figure 5C) (Beck et al., 2014b). These two CdiA-CT moieties share no function, sequence or structural homologies, reflecting how bacteria use two-partner secretion and the modular organization of CdiA proteins to deliver various kinds of toxins into target cells.

### TpsB transporters

**Figure 6:**
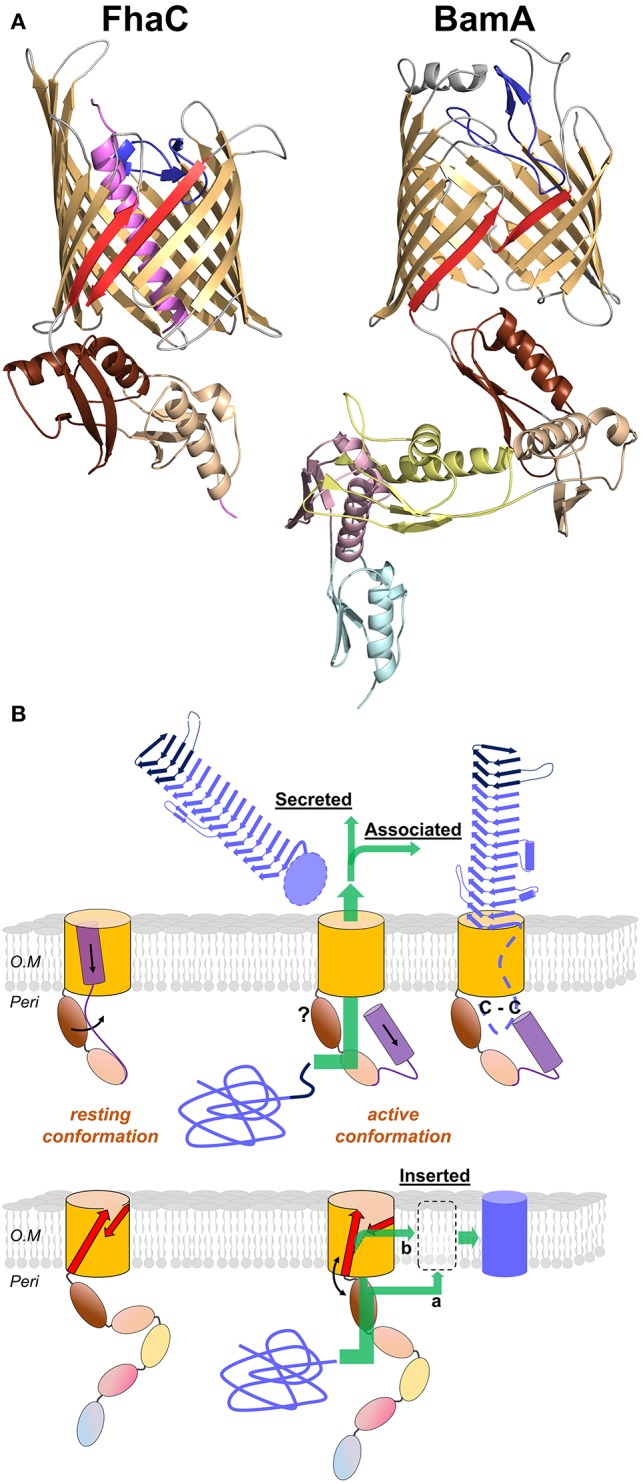
**Omp85 transporters: X-ray structures to mechanistic models. (A)** Cartoon representations of FhaC from *B. pertussis* (PDB entry 4ql0) and BamA from *N. gonorrhoeae* (4k3b). The common structural features include the 16-strand barrel in gold, the L6 loop in blue and the last two POTRA domains in wheat and brown (POTRA1 and POTRA2 for FhaC). The β_1_ and β_16_ strands are shown in red. Specific elements include the N-terminal α helix of FhaC in purple, and the POTRA3-2-1 domains of BamA in yellow, pink, and cyan, respectively. **(B)** Mechanistic models for TpsB transporters and BamA proteins (upper and lower diagrams, respectively). The substrates are colored blue, unfolded in the periplasm and folded in their final locations. For FhaC, the motions of the N-terminal α helix H1 and of the POTRA2 domain are shown with black arrows (see text). The TPS domain (dark blue) interacts with the POTRA domains, and the TpsA protein is translocated through the barrel pore (green arrow). The location of the POTRA domains at this stage remains to be determined. Depending on the subfamily, the TpsA is secreted into the milieu or remains associated with its transporter by a small globular periplasmic domain harboring a disulfide bond (C-C). For BamA, the available structures indicate at least two conformations, one in which POTRA5 is away from the β barrel and β_1_ and β_16_ are close to one another with H bonds formed between them, and another characterized by the occurrence of a lateral gate caused by the reorientation of β_1_–β_8_ and POTRA5. This gate might serve for progressive folding of the substrate through the formation of a hybrid barrel with BamA, before its release in the outer membrane (green arrow denoted b; BAM-budding model). Local destabilization of the bilayer close to the β_1_–β_16_ junction might also facilitate direct insertion of the prefolded substrate in the membrane (green arrow denoted a; BAM-assisted model). The L6 loop and the rest of the BAM complex are not represented.

The second partner in a TPS system is the TpsB protein. These proteins belong to the ubiquitous Omp85 superfamily, whose best-known members are the essential BamA transporters that assemble proteins in the outer membrane of Gram-negative bacteria (reviewed in Bos et al., 2007; Knowles et al., 2009; Hagan et al., 2011; Ricci and Silhavy, 2012). All the X-ray structures of Omp85 transporters thus far available show that these transporters share major structural features (Clantin et al., 2007; Gruss et al., 2013; Noinaj et al., 2013; Albrecht et al., 2014; Ni et al., 2014; Maier et al., 2015; Bakelar et al., 2016; Gu et al., 2016; Han et al., 2016). For TpsB transporters, the only structure reported thus far is that of FhaC (Clantin et al., 2007; Maier et al., 2015) (Figure 6A).

The C-terminal moiety of FhaC forms a 16-stranded, anti-parallel β barrel in the outer membrane, which has been shown to serve as the pore for the translocation of its FHA partner across the outer membrane (Baud et al., 2014). The β barrel is preceded by two periplasmic Polypeptide Transport-Associated (POTRA) domains in tandem, called POTRA1 and POTRA2. The POTRA domain fold is βααββ, with an anti-parallel β sheet flanked by two α helices on one side of the sheet. POTRA domains have been proposed to mediate protein-protein interactions, probably by β augmentation (Kim et al., 2007). The POTRA domains of several TpsB proteins have been shown to recognize the TPS domains of their respective TpsA partners (Surana et al., 2004; Hodak et al., 2006; Delattre et al., 2011; ur Rahman et al., 2014; Garnett et al., 2015; Grass et al., 2015). A major binding site for the FHA TPS domain in FhaC is a hydrophobic groove formed by the edge of the β sheet and the flanking α helix of the barrel-proximal POTRA2 domain (Delattre et al., 2011). This site appears to be well suited to accommodate amphipathic polypeptide segments of FHA, which interacts with FhaC in an extended, not-yet folded conformation (Hodak et al., 2006).

The β barrel of FhaC is occluded by the N-terminal α helix, H1, which crosses the barrel pore with its N terminus at the cell surface and its C terminus in the periplasm. Although not required for activity, similar helices are predicted in most TpsB proteins (Guérin et al., 2014). This N-terminal α helix might facilitate biogenesis as well as stabilize the “resting” form of the transporter (see below). The α helix H1 is followed by a 30-residue periplasmic linker in an extended conformation that joins H1 to the N terminus of the membrane-distal POTRA1 domain (Maier et al., 2015). Unlike H1, which is dispensable, the linker is necessary for FhaC secretion activity (Guédin et al., 2000; Clantin et al., 2007). The X-ray structure of FhaC has revealed that the linker occupies the FHA binding site along POTRA2. One of its functions might thus be to regulate accessibility of the binding site in a competitive mechanism (Guérin et al., 2014; Maier et al., 2015).

The β barrel of FhaC is also partially occluded by a long extracellular loop, L6, a hallmark feature of Omp85 transporters (Moslavac et al., 2005; Arnold et al., 2010). The Arg of the highly conserved VRGY/F motif at the tip of L6 forms a salt bridge with the Asp of another conserved motif, F/GxDxG, located on the β strand β13 of the barrel (Maier et al., 2015). This interaction thus positions L6 within the β barrel in a similar manner in all available Omp85 structures (Gruss et al., 2013; Noinaj et al., 2013; Albrecht et al., 2014; Ni et al., 2014; Bakelar et al., 2016; Gu et al., 2016; Han et al., 2016). Like the barrel-proximal POTRA domain, L6 is essential for the activity of Omp85 proteins. The functions of these critical pieces of the machinery remain unclear (Delattre et al., 2010; Leonard-Rivera and Misra, 2012; Rigel et al., 2013).

## Pathway of TpsA proteins across the cell envelope

The pathway of TpsA proteins from the cytoplasm to the cell surface has been reviewed in (Jacob-Dubuisson et al., 2004; van Ulsen et al., 2014). After an overview of current knowledge, we will spell out the unresolved questions on the transport mechanism.

### Export across the inner membrane and the periplasm

TpsA proteins are synthesized as preproteins, and their N-terminal signal peptide determines Sec-dependent export across the cytoplasmic membrane (Grass and St Geme, 2000; Chevalier et al., 2004). Some of them have an extended signal peptide that harbors a semi-conserved N-terminal extension (Jacob-Dubuisson et al., 2004; van Ulsen et al., 2014), which is also found in subsets of classical and trimeric AT proteins (Peterson et al., 2006; Desvaux et al., 2007; Szczesny and Lupas, 2008). Among TPS systems, the function of these extended signal peptides has been investigated only in the case of the TpsA preprotein FhaB, which is the precursor of FHA in *B. pertussis*. This study has revealed that the extension optimizes biogenesis by slowing down export and delaying signal peptide cleavage (Chevalier et al., 2004). The role of the extension has also been studied for several AT proteins, as reviewed in (Desvaux et al., 2006; van Ulsen et al., 2014). It was shown in the case of EspP that, by prolonging the association of the AT with the inner membrane, the extended signal peptide might prevent non-productive folding of the passenger domain in the periplasm (Szabady et al., 2005).

Following Sec-dependent export, the TpsA proteins reach the periplasm, where they remain in extended conformations for their interactions with their TpsB transporters. How the not-yet-folded TpsA secretion intermediates are protected from degradation, misfolding, or aggregation in the periplasm has been investigated in very few systems. Chaperone requirement by TpsA proteins might depend on their size, the proteolytic activity in the periplasm of the host bacterium, and the rates at which they tend to aggregate or to misfold. The proteins that form long β solenoids are generally slow to fold and do not have a strong tendency to aggregate, two properties favorable to Type V secretion (Hodak et al., 2006; Junker et al., 2006; Junker and Clark, 2010).

The time window during which TpsA periplasmic intermediates retain secretion competence markedly depends on the system. For the OtpAB system of *E. coli* O157:H7, expression of the OtpB transporter could be triggered after that of its TpsA partner with no detrimental effect on secretion, demonstrating that export across the cytoplasmic membrane and secretion across the outer membrane need not be temporally coupled (Choi and Bernstein, 2009). In contrast, periplasmic intermediates of FHA appeared to rapidly become secretion-incompetent *in vivo* (Guédin et al., 1998). Similarly, when secretion was reconstituted *in vitro*, delaying the addition of FhaC-containing proteoliposomes to FHA-producing spheroplasts strongly impaired translocation into the vesicles (Fan et al., 2012).

While *bona fide* periplasmic intermediates are detectable for some TpsA proteins, including ShlA, OtpA and a few others (Schiebel et al., 1989; van Ulsen et al., 2008; Choi and Bernstein, 2009), in other TPS systems, in contrast, they appear to be quickly degraded. Thus, HMW1 and FhaB were proteolyzed by the protease-chaperone DegP if their secretion was impeded, probably to limit the development of envelope stress (St Geme and Grass, 1998; Baud et al., 2009). On the other hand, in the case of FHA, DegP was also shown to facilitate secretion (Baud et al., 2009, 2011). In particular, a specific form of DegP associated with the cytoplasmic membrane was found to bind non-native FHA fragments with strong affinity, indicating that it might be a “holding chaperone” (Baud et al., 2009, 2011). The involvement of DegP has been shown in type Va, Vc, and Ve systems, suggesting that it might be general in the type V pathway (Mogensen and Otzen, 2005; Grosskinsky et al., 2007; Oberhettinger et al., 2012).

### Translocation across the outer membrane: function of the TpsB partner

Molecular recognition between the two partners is mediated by the TPS domain and the POTRA domains of the TpsA and TpsB proteins, respectively (Surana et al., 2004; Hodak et al., 2006; Delattre et al., 2011; Garnett et al., 2015). The TPS domain of the TpsA protein must be in an extended conformation for interaction with the POTRA domains of the TpsB transporter, as shown for the FHA/FhaC and HMW1/HMW1B pairs (Hodak et al., 2006; Grass et al., 2015). Surface plasmon resonance experiments with the FHA/FhaC pair have indicated interactions of micromolar affinity between the two proteins and suggested that association and dissociation occur very fast (Delattre et al., 2011). Similarly, NMR experiments on the LepAB system of *P. aeruginosa* have shown fast exchange between the bound and free forms, which also supports the idea that TpsA-TpsB interactions are dynamic (Garnett et al., 2015). While this may be expected from the fact that the partners must dissociate after secretion, this property is likely to have some bearing on the mechanisms of transport (see below). What constitutes the “secretion signal” of a TpsA protein is imperfectly defined but most likely involves a combination of conserved and non-conserved residues, as well as some extended amphipathic structure, likely for β augmentation of the POTRA β sheets (Hodak et al., 2006; Kim et al., 2007; Gatzeva-Topalova et al., 2010). Crystal structures of the soluble periplasmic domain of the Omp85 proteins BamA and TamA indicate that POTRA domains may generally interact with other proteins by β augmentation (Kim et al., 2007; Gruss et al., 2013).

Binding of the TPS domain to the POTRA domains is followed by the transport of the TpsA protein to the cell surface, most likely in an extended conformation, but a large gap persists in our understanding of the translocation step itself (Figure 6B). The path of translocation of FHA across the outer membrane has been investigated by *in vivo* cross-linking. This identified several regions of FhaC that interact with FHA: the surface of the POTRA domains, the inner lining of the β-barrel pore, and specific surface loops (Baud et al., 2014). Similarly, in particular two regions in the N-terminal and central parts of the TPS domain of FHA interact with FhaC. The interaction map reveals a funnel-like pathway, with the conformationally flexible TPS domain entering the channel in a non-exclusive manner and exiting along a specific four-stranded β sheet at the cell surface (Baud et al., 2014). Translocation initially appears to proceed in discrete steps, which is consistent with a repetitive, cyclic process (Baud et al., 2014).

Two models of translocation have been proposed. According to the first one, the TpsA protein forms a hairpin in the pore. Its TPS domain remains bound to the POTRA domains throughout the entire process, while the rest is progressively translocated and folds at the cell surface. In this scenario, the TPS domain is released at a late or final stage of translocation (Mazar and Cotter, 2006). In an alternative model, the TPS domain reaches the surface first and nucleates the folding of the rest of the protein (Hodak and Jacob-Dubuisson, 2007). Several pieces of evidence suggest that the latter mechanism is at play, notably the efficient secretion of N-terminal truncates of TpsA proteins (Jacob-Dubuisson et al., 2004), and the accessibility of the N-terminus of stalled FHA constructs at the cell surface (Guérin et al., 2014). In addition, the stable TPS fold and the observation that a TPS domain can initiate TpsA folding *in vitro* (Walker et al., 2004; Weaver et al., 2009) indicate that the TPS domain may have a similar role in initiating folding in two-partner secretion as that suggested for the stable C-terminal core of the passenger domain in autotransporter secretion (see below; Junker et al., 2006, 2009; Peterson et al., 2010; Renn et al., 2012; Besingi et al., 2013).

Irrespective of the model, the same basic questions arise: how is the TpsA protein pulled or pushed into the pore? What initiates the process and what drives it forward? Does the TpsB transporter cycle between different conformations in order to mediate the progressive transport of its substrate? It should be noted that no high-energy, hydrolyzable compounds such as ATP are available in the periplasm, and that the semi-permeable outer membrane cannot sustain an electrochemical gradient based on small ions, although a Donnan potential may exist (see below; Sen et al., 1988). The process should thus involve transitions between conformations separated by low energy barriers.

The dynamics of the FhaC transporter has been investigated using electron paramagnetic resonance (EPR), which provides information on the mobility of specific regions of the protein as well as the distances between them. These experiments revealed that FhaC exists in an equilibrium of several conformations. The first equilibrium concerns the N-terminal α helix H1 which spontaneously moves to the periplasm (Guérin et al., 2014). The movement of H1 breaks linker-POTRA interactions, thus allowing the TPS domain of the substrate protein to bind to the POTRA domains (Figure 6B). The equilibrium between the resting, closed conformation of FhaC and the open conformation of FhaC with H1 in the periplasm might thus be displaced by the binding of FHA (Guérin et al., 2014; Maier et al., 2015). Similarly, in the LepB transporter of *P. aeruginosa*, the linker may transiently interact with POTRA2 and be displaced by the substrate (Garnett et al., 2015).

The second equilibrium concerns the conserved loop L6 that is folded back inside the pore from the cell surface and interacts with the inner wall of the barrel (Guerin et al., 2015; Maier et al., 2015). Although this loop, and in particular its VRGY hallmark motif, is essential for activity (Clantin et al., 2007; Delattre et al., 2010), its function remains obscure. It does not seem to have a direct role in substrate pulling (Baud et al., 2014) and appears relatively rigid in EPR experiments (Guerin et al., 2015). Notably, in BamA, attaching L6 to the barrel does not inhibit growth, so large-scale motion of L6 does not seem to be required for BamA function (Noinaj et al., 2014). Nevertheless, the position of L6 appears to change in the pore and to modulate channel opening (Guerin et al., 2015). Although these experiments have revealed that, overall, TpsB proteins are highly dynamic, they fall short of describing the full secretion cycle.

Secretion and folding are most likely coupled in the TPS pathway. This has long been proposed to explain how secretion could proceed in the absence of classical sources of energy. Thus, once a TpsA protein emerges at the cell surface and starts to fold into a β helix, the difference in free energy between the extended and the folded forms is thought to drive secretion (Jacob-Dubuisson et al., 2004). This model has been extensively probed for type Va AT proteins whose passenger domains also fold into long β solenoids. These β helices appear to unfold and fold in several steps, and many of them appear to have a stable core that initiates folding (Oliver et al., 2003; Renn and Clark, 2008). The passenger domain of a classical AT protein forms a hairpin in the pore in the course of secretion, and thus its C-terminal portion reaches the cell surface first (Bernstein, 2007; Junker et al., 2009; Zhai et al., 2011). In many cases the C-terminal region of the passenger has a greater thermostability than the rest of the protein, and this difference is thought to promote its vectorial folding, which may then drive secretion (Junker et al., 2006, 2009; Peterson et al., 2010; Renn et al., 2012; Besingi et al., 2013). The initiation of translocation, before passenger folding can occur, may then be linked to the energetically favorable folding and membrane insertion of the β barrel, with BamA lowering the kinetic barrier (Moon et al., 2013; Gessmann et al., 2014). Consistent with the hypothesis of folding-driven secretion, mutations that impair folding of the C-terminal passenger region or the β helix in general have been observed to reduce or abolish secretion (Peterson et al., 2010; Renn et al., 2012; Drobnak et al., 2015). However, it has been noted that the free energy typically associated with protein folding is orders of magnitude smaller than the energetic cost of protein transport across a membrane as it has been measured, for example, for translocation across the chloroplast envelope (Kang'ethe and Bernstein, 2013a). Nevertheless, the possibility of reconstituting type V secretion systems *in vitro* and obtaining translocation of TpsA proteins and AT passengers into liposomes demonstrates that, in these systems, protein translocation across a membrane is indeed possible without input of external energy (Norell et al., 2014). One solution that has been proposed to this apparent conundrum is a “Brownian ratchet”-type mechanism in which random thermal motion obtains directionality via an effectively irreversible step, here, the folding of the transport substrate on the extracellular side, which prevents backtracking of the protein chain (Peterson et al., 2010; Drobnak et al., 2015). Apart from the folding free energy, the required free energy for this process would be contributed by the mechanisms that keep secretion substrates unfolded in the periplasm, i.e., most likely chaperones (Baud et al., 2009; Jacob-Dubuisson et al., 2013). Such mechanisms have been described theoretically (Simon et al., 1992; Depperschmidt et al., 2013) and demonstrated experimentally e.g. for transport into the endoplasmatic reticulum (Matlack et al., 1999), suggesting that they may also be involved in bacterial type V secretion.

Still, it has been observed that, when engineered into an AT sequence, even a disordered domain that does not assume a stable structure could be secreted. This occurred even if the remaining passenger domain fragment was too short to fold (Kang'ethe and Bernstein, 2013b). A Brownian ratchet mechanism thus cannot be at play in this case. However, removal of negatively charged residues in the passenger was observed to inhibit secretion in this system. In this context, it is interesting to note that sizable Donnan potentials—with the periplasmic side negative—have been observed across the *E. coli* outer membrane and traced to the presence of negatively charged oligosaccharides (Sen et al., 1988). The observation that a disordered, negatively charged polypeptide can be secreted thus suggests that an electrochemical potential based on large, impermeable charged molecules may indeed be present across the bacterial OM and play a role in type V secretion. In addition, the process might also benefit from entropic effects due to the difference between the crowded periplasmic environment and the extracellular milieu (Fan et al., 2016); however, extracellular crowding agents have not been observed to affect AT secretion (Drobnak et al., 2015).

The same principles of differential stability between distinct regions of the secreted β helix and of vectorial folding observed in AT proteins likely apply to TPS systems as well, and therefore the region emerging first from the TpsB channel probably nucleates folding of the β helix at the cell surface. The conserved TPS domain is an obvious candidate for this function, and several data support the idea. Early studies have indicated that the TPS domain of ShlA was able to template the folding of the unfolded full-length protein to form the active toxin *in vitro* (Schiebel et al., 1989; Schönherr et al., 1993). Similar experiments with the TPS domain of HpmA have shown that cooperative β strand interactions mediate the folding of neighboring full-length proteins, a process called “template-assisted hemolysis” (Weaver et al., 2009). Two more recent pieces of work have addressed the unfolding properties of the TPS domains of FHA and HpmA, by using atomic force microscopy (Alsteens et al., 2013) or chemical denaturation (Wimmer et al., 2015), respectively. Both studies have shown that TPS domains unfold in several steps and harbor highly stable core subdomains. In the second case, the presence of the folded core subdomain allowed rapid folding of the rest of the TPS domain *in vitro*. It is tempting to speculate that these core regions of the TPS domain fold early in the course of secretion and nucleate folding of the rest by β augmentation concomitant with translocation, and that a Brownian ratchet-type of mechanism is at play in TPS secretion as well.

Translocation initiation in TPS systems cannot be linked to the free energy gained from β barrel folding and membrane insertion, as has been proposed for AT proteins (Moon et al., 2013), since the two processes are independent. Here, the specific but transient interactions observed between the TpsB transporter and the TPS domain of its substrate likely play a role (Delattre et al., 2011).

The possibility that the TpsB transporter assists in the initial folding of its partner has been suggested by recent observations in two different TPS systems. Thus, specific interactions were shown to occur between parts of the FHA TPS domain and specific surface β strands and loops of FhaC (Baud et al., 2014). The edge of this small surface-exposed β sheet of FhaC might guide the secretion of the TPS domain and template its initial folding at the cell surface by β augmentation (Baud et al., 2014). Interestingly, the corresponding loops of HMW1B appear to tether the HMW1 adhesin at the cell surface of *H. influenzae* (Grass et al., 2015). Thus, specific structural features at the extracellular surface of the TpsB transporter might be involved in the secretion of the cognate TpsA partner and possibly also retain its N terminus close to the cell surface.

### What can we learn for the TPS pathway from the role of BamA in type V secretion?

TpsB transporters are Omp85 proteins specialized in the transport across the outer membrane of soluble protein substrates (Heinz and Lithgow, 2014). In parallel with the role of a specialized Omp85 transporter for type Vb secretion, there have been multiple demonstrations that another Omp85 transporter, BamA, and indeed the BAM complex, is involved in the folding and insertion of most outer membrane proteins, including classical ATs (type Va) (Jain and Goldberg, 2007; Ieva and Bernstein, 2009; Sauri et al., 2009; Ieva et al., 2011), trimeric ATs (Lehr et al., 2010) and type Ve intimins (Bodelón et al., 2009; Oberhettinger et al., 2012). Omp85 transporters thus form the mechanistic basis of type V secretion.

In spite of the many structures now available of BamA and the BAM complex (Figure 6A), our models for the molecular mechanisms of protein assembly in the outer membrane remain somewhat speculative (Hagan et al., 2011; Noinaj et al., 2015). Several studies have shown that BamA opens laterally between its first and last β strands in the outer membrane and that this opening is required for function (Noinaj et al., 2013, 2014; Albrecht et al., 2014; Iadanza et al., 2016). Another important feature common to all BamA structures is a decreased hydrophobic thickness where the first and last β strands meet, which causes a local thinning of the outer membrane (Noinaj et al., 2013, 2014). Two popular mechanistic models for BamA-mediated protein assembly are the BAM-assisted and BAM-budding models (Figure 6B). It should be noted that they have not been experimentally proven, and it is possible that different models apply to different classes of OMPs. The BAM-assisted model postulates that some OMPs can fold without help from accessory proteins and need only a locally destabilized outer membrane in which to insert the fully folded OMP. BamA locally disturbs the outer membrane where its first and last β strands meet, and it could serve as a folding catalyst by lowering the kinetic barrier to nascent OMP insertion (Burgess et al., 2008; Noinaj et al., 2013, 2015; Gessmann et al., 2014). The BAM-budding model takes into account the requirement for lateral opening of BamA and might explain how BAM helps to fold OMPs that do not spontaneously fold on their own. In this model, an unfolded OMP would be delivered to the BAM complex by periplasmic chaperones such as SurA and Skp. The nascent OMP would then be sequentially folded by pairing individual β strands with the first and last β strands of BamA separated by the lateral opening. In this model, β strands from the nascent OMP would be added to BamA, forming a hybrid BamA-OMP barrel, and eventually the new OMP would separate from BamA and move away laterally into the outer membrane (Figure 6B).

Classical AT proteins (type Va) are composed of both a β barrel moiety and a soluble passenger domain, and several pieces of work have indicated that the BAM complex is involved in both the insertion of the β barrel moiety into the membrane and the translocation of the passenger domain to the bacterial surface (Ieva et al., 2008; Ieva and Bernstein, 2009; Sauri et al., 2009). For the AT protein EspP, an assembly intermediate was detected in the periplasm whose topology resembled that of the assembled protein, with a segment preceding the barrel moiety embedded in a “proto-barrel” (Ieva et al., 2008). Interactions were detected between the BAM complex and both the barrel and the passenger domains of the AT substrate (Ieva and Bernstein, 2009). Formation of the β barrel domain thus appears to proceed to a certain extent in the periplasm, with a β hairpin that corresponds to a segment C-terminal of the passenger incorporated in its pore. According to these authors, the proto-barrel would then undergo a conformational change before translocation is initiated. The passenger domain would interact with the POTRA domains of BamA and progressively move through a channel, composed of BamA and/or the AT barrel (Pavlova et al., 2013). These observations are at odds with the model that the β barrel of the AT protein assembles progressively, hairpin by hairpin, in the outer membrane as suggested by the budding model, and they argue that the lateral opening of the BamA barrel might play another role in the assembly/secretion process of ATs.

What about TPS systems? TpsA proteins are soluble and have no transmembrane domain, and thus they represent “simpler” substrates than AT proteins. *In vitro* reconstitution of TPS secretion has shown that, while TpsB proteins are inserted into the OM by the BAM complex like most outer membrane proteins (Norell et al., 2014), they form autonomous secretion ports for their TpsA substrates (Fan et al., 2012). In other words, the translocation of a TpsA protein only requires its TpsB partner and does not proceed through a channel also involving BamA. A TpsB transporter thus mediates the translocation of its soluble TpsA substrate by itself.

The role of the POTRA domains in Omp85 transporters is likely to go beyond the recruitment of substrate proteins, as suggested by a point mutation in POTRA2 that affects the function of FhaC but not the binding of FHA (Delattre et al., 2011). This mutation changes a highly conserved Asp residue in the TpsB family that participates in periplasmic interactions between the POTRA2 domain, the linker and the barrel (Maier et al., 2015). The POTRA domains might thus contribute to addressing the TpsA protein to the pore of the TpsB transporter. Similar suggestions were made for the POTRA domains of HMW1B and LepB (Garnett et al., 2015; Grass et al., 2015). Their role in shuttling the passenger to the barrel might explain why PlpD, the prototypic Vd protein, harbors a POTRA-like domain. Since the Patatin domains of the passenger are part of the same polypeptide as the TpsB-barrel-like transporter moiety, the POTRA-like domain is not necessary to recruit the substrate.

Both BamA and TpsB transporters most likely work in a cyclic manner and go through their “conformational cycle” several times in the course of secretion of their long substrates (Figure 6B). The conformational changes that they undergo remain to be described, but we propose that they involve concerted motions of the three conserved structural components essential for activity, i.e., the β barrel, the L6 loop and the barrel-proximal POTRA domain. A few observations support this hypothesis. It has been proposed that the L6 loop of BamA cycles between several conformations, and that its conformation is modulated by other Bam proteins via the POTRA5 domain (Rigel et al., 2013). A functional connection between the latter and L6 in BamA has also been found in a search for intragenic suppressors of a mutation that disrupts the L6-barrel interaction (Dwyer et al., 2013). Functional links between L6 and the barrel are clearly established. For instance, the FhaC channel becomes larger if the interaction between L6 and the wall of the barrel is disrupted (Guerin et al., 2015). Similarly, two conductance states have been recorded for the HMW1B transporter. The higher-conductance state is occasionally visited by the protein and likely corresponds to a more open channel (Duret et al., 2008).

Our current hypothesis for the mechanism of two-partner secretion is that the TpsB barrel cycles between narrow-channel and open-channel states. As seen with BamA, the conformational changes might induce a reorientation of β1 and POTRA2, with the formation of a lateral opening between the first and last strands of the β barrel. This lateral opening could be used to enlarge the β barrel with the insertion of amphipathic β hairpin(s) from periplasmic portions of the protein, thus hoisting a first portion of substrate bound to the POTRA domains toward the surface. After the substrate is released on the extracellular side of the outer membrane to initiate folding, possibly along a template provided by extracellular parts of the barrel, the inserted region switches back to the periplasm to repeat the cycle. We hypothesize also that L6 can stabilize these conformations by forming distinct interactions with specific regions of the β barrel in each of the two states. The TpsA protein might thus “hitch-hike” on spontaneous conformational changes of its transporter for translocation. Together with extracellular-only folding as required for a Brownian ratchet mechanism, and possibly a Donnan potential, our proposed mechanism accounts for the vectorial translocation of TpsA proteins across the bacterial OM.

## Conclusion

TPS systems are widespread among Gram-negative bacteria, both pathogenic and environmental. They serve many different functions that participate in the interactions of the bacteria with their environment, be it the host cell or bacterial competitors. The diversity of type V systems deployed by bacteria is probably yet larger than we have come to realize, with hybrid systems made up of parts borrowed from a variety of protein domains. The mechanisms of transport of such diverse and sometimes extremely long domains remain poorly understood. Endeavors to decipher those mechanisms will undoubtedly illuminate the inner workings of Omp85 transporters.

## Author contributions

All authors listed have made substantial, direct and intellectual contribution to the work, and approved it for publication.

### Conflict of interest statement

The authors declare that the research was conducted in the absence of any commercial or financial relationships that could be construed as a potential conflict of interest.
